# Hysteresis in motor and language production

**DOI:** 10.1177/17470218221094568

**Published:** 2022-05-18

**Authors:** Amy L Lebkuecher, Natalie Schwob, Misty Kabasa, Arella E Gussow, Maryellen C MacDonald, Daniel J Weiss

**Affiliations:** 1 Department of Psychology, The Pennsylvania State University, University Park, PA, USA; 2 Department of Psychology, University of Wisconsin–Madison, Madison, WI, USA

**Keywords:** Hysteresis, priming, motor planning, language production, plan reuse

## Abstract

*Hysteresis* in motor planning and *syntactic priming* in language planning refer to the influence of prior production history on current production behaviour. Computational efficiency accounts of action hysteresis and theoretical accounts of syntactic priming both argue that reusing an existing plan is less costly than generating a novel plan. Despite these similarities across motor and language frameworks, research on planning in these domains has largely been conducted independently. The current study adapted an existing language paradigm to mirror the incremental nature of a manual motor task to investigate the presence of parallel hysteresis effects across domains. We observed asymmetries in production choice for both the motor and language tasks that resulted from the influence of prior history. Furthermore, these hysteresis effects were more exaggerated for subordinate production forms implicating an inverse preference effect that spanned domain. Consistent with computational efficiency accounts, across both task participants exhibited reaction time savings on trials in which they reused a recent production choice. Together, these findings lend support to the broader notion that there are common production biases that span both motor and language domains.

In the mid-20th century, Lashley (1951) argued against a prevailing theory that sequential behaviours could be best explained by simple associative chaining (e.g., Washburn, 1916). He proposed that serial actions in behaviour, such as language production and complex motor sequences, arise as a consequence of hierarchical planning (see Rosenbaum et al., 2007). Despite this theory gaining relatively broad acceptance (though see, e.g., Wickelgren, 1969), research on language and action planning has remained largely siloed within their disciplines. While there have been debates regarding the extent to which nonlinguistic action sequences share *structural* properties with language (e.g., Jackendoff, 2011; Pulvermüller, 2014), there is a dearth of empirical data to support the existence of shared cognitive mechanisms underlying planning in each domain.

One approach to aligning action sequencing and language production has been to describe a “grammar of action” that is analogous to syntax in language. A variety of claims have been made that the consistent rules and patterns governing motor production can be described as hierarchically organised compositional systems akin to grammar in language. Examples of grammatical organisation have been drawn from a variety of actions, such as drawing (Goodnow, 1977; Goodnow & Levine, 1973), eye movements (Juhola, 1995), and tool use (Pastra & Aloimonos, 2012). While theorising about a grammar of action is certainly consistent with the aims of the current study, we note that these efforts tend to be limited. Specifically, the design of action grammars tends to follow contemporary models of syntax, rather than building from emergent properties of action that might depart from linguistic counterparts. On that view, the grammar of action, thus far, has served to translate action sequences in terms created for linguistic constructs, rather than promoting serious dialogue between the two disciplines.

Several empirical approaches have been employed in an effort to characterise the apparent parallels between serial ordering in language production and nonlinguistic domains. One approach has focused more on the possibility of shared resources by exploring whether it is possible to prime from one domain to another, as in language and music (e.g., Koelsch et al., 2004), or mathematical computations (e.g., Allen et al., 2010; Koelsch et al., 2004; Pozniak et al., 2018; Scheepers et al., 2019; Van de Cavey & Hartsuiker, 2016). Another approach has been to search for common effects that span the production and recall of planned sequences across different domains (e.g., Hurlstone et al., 2014), such as common error patterns in speech and piano playing (Palmer & Pfordresher, 2003). Similarly, research from our laboratory groups discovered that expert jazz musicians conform to a sequencing bias described in language production (Easy First; see MacDonald, 2013) when improvising (Beaty et al., 2021). Such parallels have also been reflected to some extent in the design of several computational models (e.g., Kello & Plaut, 2004). Together, these lines of research all point to the possibility that language sequencing shares important characteristics and possible cognitive resources, with nonlinguistic domains, consistent with Lashley’s (1951) initial observation.

Consequently, the goal of this study is to investigate commonalities in language and action planning by implementing tasks that share similar design characteristics in each domain. Given the divergence in methods used for studying language and action, one way to bridge these research areas is to create parallel tasks that adopt established methods from one domain and implement them in the other. For example, Koranda and colleagues (2020) presented participants with a syntactic priming task and an analogous motor task designed to elicit reuse in action production. The language task involved describing a scene using one of two-word-order patterns and the motor task required touching dots on a screen using one of the two possible sequences. Motor and language trials were intermingled within an experimental session. There were three general findings from this work. As expected, within each domain, the primes were effective as would be predicted based on prior research within each discipline. Moreover, exploratory analyses provided some preliminary evidence for priming across domains suggesting some common processing. Finally, the authors identified the central challenge for this type of approach, which is balancing the task demands across domains in terms of difficulty (see Koranda et al., 2020).

The current study builds on this approach in a novel and complementary way. To the best of our knowledge, this study represents the first attempt to design a phrase-ordering language task based on principles derived from the literature on motor planning. Similar to action planning paradigms, the target stimuli on the language task in the current study change incrementally on a trial-by-trial basis. This is radically different from standard methods in language research that contain a comparatively small number of experimental trials separated by many filler trials. The filler trials are generally not analysed further but are designed to obscure the experimental manipulation and also to discourage effects of repetition from one trial to the next. These repetition effects are exactly the target of investigation here, and our use of this design amounts to a claim that we will be able to observe similar effects of production history in both the language and action tasks with incremental changes from one trial to the next.

The focus of our study is on *hysteresis*, a term commonly used across multiple disciplines (e.g., physics, chemistry, kinesiology) to describe the impact of prior history on the state of a physical system. With respect to motor control, hysteresis refers to the effects of recently executed actions on current executions (Haken et al., 1985; Kelso, 1981; Valyear et al., 2018). A classic example of hysteresis in this domain involves rhythmic finger movements. The point of switching (i.e., transition point [TP]) between in-phase and anti-phase muscle contractions is asymmetric; varying as a function of whether individuals are speeding up or slowing down their movements (Kelso, 1981). This delay in the transition between in-phase and anti-phase muscle contractions as a function of prior movement history is a hallmark of hysteresis. Such effects have often been explained without invoking cognition per se, such as dynamical system approach, that consider hysteresis as an emergent property of biomechanical considerations and task-specific attractor states (Haken et al., 1985; Kelso, 1981).

Contrasting this approach, cognitive models of motor planning, such as posture-based motion planning (Rosenbaum et al., 2001, 2006; Rosenbaum & Jorgensen, 1992), suggest that hysteresis-like asymmetries in production arise because individuals must quickly decide between competing alternative plans stored in memory during action production (Wolpert & Landy, 2012). According to this model, a goal posture (e.g., the use of a specific hand and grasp to complete an action) is first selected from memory and then adapted to suit the demands of the current environment. Operating within this framework, hysteresis can be described as the selection of a recently activated goal posture, consistent with the view that both cognitive and biomechanical factors are relevant.

Correspondingly, the cost-optimisation hypothesis proposes that the total cost of a movement is a combination of the cognitive cost of motor planning and the mechanical cost of motor execution (Schütz et al., 2016). In a similar vein, Valyear and colleagues (2018) developed the computational efficiency model of action hysteresis proposing that hysteresis effects are more prevalent when the biomechanical costs of competing action plans are roughly equivalent. The computational efficiency model is supported by the observation that reusing the same hand in reaching task results in shorter reaction times (RT) relative to switching hands, reflecting the benefits of plan reuse relative to generating a new motor plan (Valyear et al., 2018). Moreover, the shorter RT associated with hand reuse is correlated with reduced activation in cortical areas involved in action planning (e.g., bilateral posterior parietal cortex and right-lateralised parietal operculum; Valyear & Frey, 2014, 2015). This cognitive view of motor planning has received further support from studies that find concurrent motor planning or execution can eliminate recency effects in verbal recall (e.g., Schütz & Schack, 2020b; Weigelt et al., 2009).

In language production, similar reuse effects have been discussed, often using the terms priming or persistence. *Syntactic priming* and *structural persistence* refer to the tendency to produce a novel sentence using a recently activated syntactic structure (Bock, 1986). For instance, after producing or hearing a sentence with prepositional phrase (PP) structure (e.g., *The server brought the food to the customer*), a speaker is more likely to describe a picture depicting a novel scene using similar phrasing (e.g., *The boy gave a gift to the girl*) relative to an alternative that does not contain a PP (e.g., *The boy gave the girl a gift*). Syntactic priming effects are relatively ubiquitous and have been observed in children (Foltz et al., 2015; Garraffa et al., 2015; Huttenlocher et al., 2004; Kidd, 2012) and even across languages in bilingual speakers (Bernolet et al., 2013; Hartsuiker et al., 2004; Schoonbaert et al., 2007).

Unlike hysteresis effects in motor control, syntactic priming has generally been attributed to cognitive efficiency biases rather than biomechanical factors (see Koranda et al., 2020). A key argument against a primarily biomechanical locus of syntactic priming is that the phenomenon can occur even when producers encounter the prime without producing it themselves (e.g., Bock et al., 2007; Chang et al., 2000). Moreover, most planning of word orders is thought to occur prior to consideration of biomechanical factors involved in articulation, and thus the priming effects would also precede later biomechanical processes (see Koranda et al., 2020; McDonald et al., 1993) for which parallel planning effects have already been demonstrated (e.g., Rosenbaum et al., 1986). Similar to the RT savings found in hysteresis effects in the motor domain (Valyear et al., 2018), reusing the same syntactic structure in language production also yields RT savings relative to producing a different structure (Smith & Wheeldon, 2001). Likewise, speakers produce words at a faster rate when repeating sounds and syllables relative to novel productions (Sevald & Dell, 1994; Sevald et al., 1995). These similar RT savings observed in both the motor and language planning literature support the assertion that individuals exhibit a bias for the reuse of previously activated production plans, or *Plan Reuse* (MacDonald, 2013) because it is less costly than generating a new plan from scratch (Rosenbaum & Jorgensen, 1992; Valyear et al., 2018) and leads to more efficient production.

## The present study

To investigate parallel hysteresis effects, we designed motor and language production tasks that involved similar incremental changes across trials. Our motor task was adapted from a previous study investigating hysteresis in the reaching behaviours of cotton-top tamarin monkeys (*Saguinus oedipus*; Weiss & Wark, 2009). In that experiment, tamarins reached through a hole in a sheet of Plexiglas to retrieve marshmallows placed at 1 of 11 fixed locations in an arc of equidistant points around the aperture. Each trial, the position of the marshmallow was incremented by one location such that across trials, the marshmallow progressed in either a clockwise or counterclockwise arc from the perspective of the subject. The tamarins tended to persist with using their initial hand for positions past the midline in both directions before switching. By comparing the locations at which individuals switched hand use (i.e., the TPs) when completing the task in the clockwise versus counterclockwise progressions, it was possible to identify a hysteresis area, an area of presumed uncertainty in which subjects were more likely to switch hands (see also Rostoft et al., 2002; Schütz & Schack, 2020a). For our study, human participants reached through a metal hoop aperture to touch dots as they appeared on a touchscreen. Similar to the paradigm in Weiss and Wark (2009), 11 targets were spaced equidistantly at fixed locations in an arc configuration. The targets appeared one at a time, progressing incrementally clockwise or counterclockwise from the perspective of the participant. We expected to observe an asymmetry in the TP as a function of prior history, similar to Weiss and Wark (2009), revealing a hysteresis area in hand use.

For the language task, we modified an experimental paradigm designed to evaluate the rates of production of a sentence type called Heavy Noun Phrase Shift (HNPS; Stallings et al., 1998). HNPS is the tendency for speakers to produce long or “heavy” noun phrase (NP) direct objects at the end of a verb phrase rather than immediately following the verb (Stallings et al., 1998; Wasow & Arnold, 2003). For example, in utterances that consist of a subject-verb phrase, a direct object NP, and a PP, English speakers tend to prefer to place the object NP before the PP (e.g., *I explained the plans to Michael*) even though the alternative (e.g., *I explained to Michael the plans*) is also acceptable in English. However, with very long object NPs, speakers become more likely to produce the “shifted” form with the PP-first (as in *I explained to Michael the extravagant plans for the new amusement park near the interstate*). This pattern has been attested in both spontaneous speech and written corpora (Hawkins, 1994; Wasow & Arnold, 2003) and in speech elicitation experiments in the laboratory (Stallings et al., 1998).

HNPS is thought to occur as a result of an *Easy First* bias, the tendency for speakers to place more easily retrieved words and phrases earlier in an utterance to alleviate the cognitive demands associated with language production (MacDonald, 2013; but see Hawkins, 1994, 2003 for a different interpretation). While several factors may contribute to HNPS structures, there is evidence that the propensity for HNPS in the production of English utterances increases with the length of the NP in words. For our purposes, the key features of HNPS are first that it affords two viable response options (phrase orders), and that the tendency to produce a PP-first form increases with the difference in word length between the PP and the NP (Hawkins, 1994; Stallings & MacDonald, 2011). The phenomenon of HNPS thus creates an opportunity to design a language task in which behaviour (use of the NP-first or PP-first form) may be expected to vary on a continuum.

We created a language task in which the NP weight was incremented, analogous to the target progression in the motor task. This methodology was loosely based on an experiment by Stallings and colleagues (1998) who presented participants with three phrases on a computer screen, including a two-word PP, and a short (2-word) or long (10-word) NP. In that study, participants were significantly more likely to produce the PP before the NP when the NP was long relative to when it was short. Based on linguistic corpus data, it has been argued that speakers become more likely to shift NP direct objects to the end of an utterance when the length of the NP exceeds the other constituent in the predicate (in this case, the PP) by four or more words (Hawkins, 1994). Given the possibility of a critical value for HNPS, evaluating this effect using an incremental procedure might allow for the discovery of a hysteresis area, which could manifest as a series of NP lengths in which production choices might differ as a function of prior history.

While both our language and motor tasks manipulated prior production history by implementing an incremental design, it is important to bear in mind the difficulties associated with creating parallel tasks across these domains (see Koranda et al., 2020). Consequently, there were design features that were not perfectly balanced across tasks. For example, the motor task, unlike the language task, had overt biomechanical constraints, such that hand use in the extreme positions would likely be uniform, as observed in previous studies (e.g., Rostoft et al., 2002; Valyear et al., 2018; Weiss & Wark, 2009). By contrast, syntactic priming is thought to occur prior to the involvement of biomechanical factors (see Koranda et al., 2020). Thus, the language task was not designed to manipulate biomechanical factors. Consequently, production choices at the extremes of the continuum were expected to be more variable. For example, previous studies of HNPS suggest that participants produce 40%–50% PP-first phrasing on trials with long NPs (Hawkins, 1994; Stallings et al., 1998; Stallings & MacDonald, 2011; Wasow & Arnold, 2003).

An additional challenge in balancing the tasks includes the amount of variability in stimulus features. While only the position of the target varied from trial-to-trial in the motor task, the words comprising the phrases on the language task differed between trials (see Table 1 in the “Methods” section). This variability was necessary to obscure the nature of the task and reduce the use of explicit strategies for sentence creation. In sum, our tasks had a common incremental feature and tested Plan Reuse, but due to differing constraints and considerations across domains, there were also differences that could impact how hysteresis would manifest in each task and correspondingly with the analytic approaches for analysis.

**Table 1.** Example stimuli for increasing (I) and decreasing (D) trials in the increasing-first condition.

SVP	NP	PP	Trial type
I described	the evidence	to Janet	I
I suggested	a blue sofa	to Linda	I
I announced	a new Vice Chair	to Carol	I
I mentioned	the recent improvements in processing	to Jacob	I
I confessed	the story on the steering defects	to Tracy	I
I recommended	some toys, winter clothing, and snow boots	to Wanda	I
I muttered	the schedule for a suspense film and budget	to Michelle	I
I stated	their strong preference for a spiral cut ham over turkey	to Michael	I
I explained	the answers on the twenty-point quiz on English Literature	to Victor	I
I proposed	a less complicated solution that had no mistakes and fewer steps	to Robert	I
I indicated	the next safest route to avoid the long and winding highway detour	to Mary	I
I proposed	an exciting alternative to their usual Friday night activity of bowling	to Brenda	D
I explained	the most important facts related to import and export taxes	to David	D
I stated	their new goal for the reduction of healthcare costs	to Lucas	D
I muttered	the plan for the poster and science fair	to Brian	D
I recommended	more specific plans for the county buildings	to Peter	D
I confessed	the secret plans for the defence missiles	to Matthew	D
I mentioned	some flyers for the lectures	to Julie	D
I announced	the math exam results	to Kendra	D
I suggested	a large package	to Jamie	D
I described	each point	to Tyler	D

Irrespective of these differences, the primary focus of our analyses was investigating production choice asymmetries as a function of prior production history. We also hypothesised that the likelihood of reuse in production might vary as a function of response preference. A well-established feature of priming on syntactic choices, termed the *inverse preference effect* (e.g., Ferreira & Bock, 2006; Segaert et al., 2016), suggests that there are more robust priming effects for less-frequent sentence structures. Evidence for this effect can be found at many levels of language use, including syntactic choices (Dell & Chang, 2014; Ferreira & Bock, 2006; Haskell et al., 2010; Jaeger & Snider, 2013; Reitter et al., 2011; Scheepers, 2003; though see Koranda et al., 2020). Thus, if subordinate plans lead to greater priming effects in both language and motor tasks, our findings would provide further evidence for parallels across domains. Finally, we were also interested in determining whether there were RT benefits for plan reuse in both tasks, as would be predicted by computational efficiency accounts of hysteresis (e.g., Rosenbaum & Jorgensen, 1992; Valyear et al., 2018).

## Methods

### Participants

Participants were 205 undergraduate students recruited from participant pools at Penn State University and University of Wisconsin-Madison. We excluded subjects who were non-native speakers of English (*N* = 7) or not right-hand dominant (*N* = 30). This left us with a sample of 165 participants (33 males and 125 females) whose average age was 18.82 years (*SD* = 1.08). Participants were classified as non-right-hand dominant if their laterality quotient on the Edinburgh Abbreviated Handedness Inventory was less than 61 (Veale, 2014). For the analysis of the motor task, we excluded subjects who did not switch hands during the arc progression (*N* = 5) as we were interested in comparing TPs (this is consistent with prior studies of hysteresis in reaching, see Weiss & Wark, 2009). For similar reasons, in the language analyses, we excluded participants who never switched phrasing order during the language task (*N* = 34).

### Motor task

#### Materials

The motor task was completed on a Dell Optiplex 9020 desktop computer with a 23-inch touchscreen monitor laid flat on a table. The task was designed and run using PsychoPy version 1.8.3.4 software (Peirce et al., 2019). The aperture for participants to reach through during the task was constructed of pliable metal and wood with a hoop portion approximately 6″ inches in diameter and was attached to a fixed location on the desk. Participants were also provided an office chair with adjustable height to sit in during the task. Videos of participants’ performance during the task were recorded with a Panasonic HC-V700M video camera.

#### Stimuli

**Figure 1.**
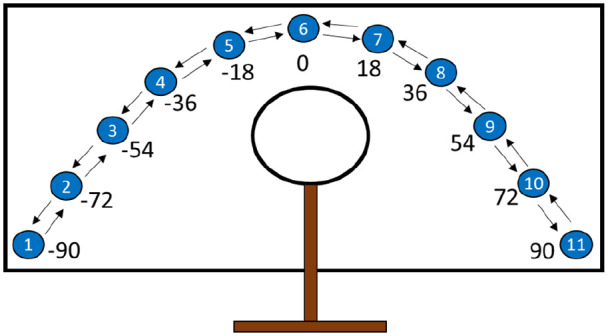
Diagram illustrating the full arc of targets for the motor task. *Note.* The target dots appeared one at a time. The arrows illustrate directions of the two arc progressions. The numbers inside the target dots are shown to illustrate the position of the target, and the numbers below are the angle of each target in degrees relative to the midline. The arrows and numbers were not visible during the experiment. The computer monitor rested flat on a table and participants reached through the hoop to touch dots on the screen. Rather than parallel to the touchscreen as shown, the hoop aperture was positioned perpendicular to the touchscreen with the flat base on the desk and the hoop portion on top.

Here, 16 blue dots (5 for practice trials and 11 for the main task) and 50 pixels each in diameter appeared on the touchscreen one at a time. Dots for practice trials appeared in five fixed locations: 0°, −36°, 36°, −90°, and 90° relative to the midline. Negative values indicate a location to the left of the midline, while positives indicate a location to the right. The 11 dots for the main task appeared one at a time in arc at −90°, −72°, −54°, −36°, −18°, 0°, 18°, 36°, 54°, 72°, and 90° relative to the midline (see Figure 1). The main task progressed through the arc one position at a time clockwise or counterclockwise.

#### Task

Before beginning the task, the experimenter instructed participants to adjust the chair height to their comfort level. The experimenter verified the adequacy of the chair height by asking the participant to reach through the hoop aperture and touch all four corners of the touchscreen monitor. Participants were then instructed (both by the experimenter and by onscreen text) to hold down the “F” and “J” keys with their left and right index finger, respectively, until a dot appeared on the screen. They were informed that the task would involve reaching through the hoop with one hand to touch dots as they appeared on the screen while continuing to press down on the appropriate key with their other hand. After touching a dot, the participant returned that hand to the “F” or “J” key and continued to press down until the next trial. The experimenter made it clear to participants that they should feel free to use either hand during the task.

Before test, there were five practice trials where dots appeared at fixed points (not in an arc configuration as in the trials on main task) on the screen as described in the previous section. The practice trials were designed to familiarise participants with the task and encourage the use of both hands. The main part of the motor task consisted of four arcs of 11 dots appearing as described in the previous section. Participants first completed two arcs in succession. The first arc followed either the clockwise or counterclockwise progression with the order counterbalanced across participants. During the second arc, participants completed the progression opposite the one completed in the first arc. Following the second arc, participants engaged in a number of intervening tasks. Participants returned to the motor task to complete the third and fourth arcs at the end of the experimental session. For the third and fourth arcs, the order of dots appearing (clockwise or counterclockwise) was reversed (e.g., if the participant initially completed the task going clockwise in the first arc and then counterclockwise in the second, the third arc started counterclockwise, and the fourth arc was clockwise). All trials were video-recorded to ensure accurate data coding.

### Language task

#### Materials

Participants completed the task on a Dell Optiplex 9020 desktop computer with a standard 24″ widescreen monitor. The task was run using PsychoPy version 1.8.3.4 software (Peirce et al., 2019). The participants’ speech was digitally recorded with a Blue^©^ Snowball iCE microphone.

#### Stimuli

**Figure 2.**
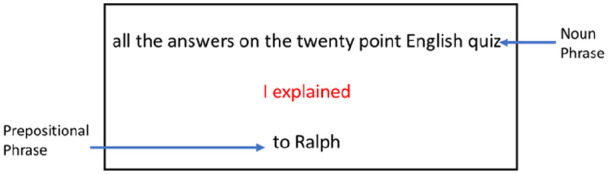
Sample display viewed by participants during the language task. *Note.* The arrows and labels “Noun Phrase” and “Prepositional Phrase” were not shown to participants but are included here for explanation of the stimuli.

Each display consisted of three phrases, with one phrase each at the top, centre, and bottom of the display (see Figure 2). The initial subject-verb phrase (e.g., “I explained”) was always in the centre in red, while the NP and PP were displayed at the top and bottom of the screen (location counterbalanced across trials). These displays were generated pseudorandomly for each trial. The initial subject-verb phrase always had the subject “I” paired with 1 of 11 possible verbs. The PP always consisted of 1 of 21 possible phrases beginning with “to” and ending in a person’s name. To provide a wide variety of stimuli within and between subjects, 242 possible NPs were generated that ranged in length from 2 (e.g., “the answers”) to 12 words (e.g., “the answers to last week’s challenging twenty-point quiz on English Literature”). Each participant viewed only 21 of the 242 possible NPs. This ensured that effects due to differences in the length of the NP did not occur as a result of any particular NPs. A subset of 21 phrases was selected to allow participants to complete one or two trials for each NP weight (2–12 words in length). The logic of the 21 trials is explained in greater detail in the next section.

#### Task

Participants were instructed to create sentences out loud using all phrases on the screen, always starting with the phrase in the centre and ending with both of the other phrases in whatever order they preferred. They were presented with 21 trials that increased and decreased in NP length in a single session (see Table 1). Increasing trials began with a stimulus screen that had a two-word NP which was subsequently increased by one word per trial until reaching a maximum length of 12 words. Decreasing trials began with a 12-word NP and decreased by one word per trial until reaching a minimum length of two words.

All participants completed both increasing and decreasing trials, but some participants began with increasing trials (*Increasing-first* condition) while others began with decreasing trials (*Decreasing-first* condition). Every participant regardless of condition completed two trials for NPs ranging from 3 to 11 words. Participants in the Increasing-first condition also completed two trials of two-word NPs, but only one trial with a 12-word NP. Those in the Decreasing-first condition completed two trials with 12-word NPs, but only one trial with a two-word NP. Due to the incrementally increasing and decreasing nature of the task, these adjustments were made by condition to ensure that no two adjacent trials had the same NP length. The task was self-paced, and participants pressed the space bar to initiate each trial. All responses were audio-recorded for data coding purposes.

### Questionnaires

Paper and pencil versions of the Language History Questionnaire (LHQ; Li et al., 2006) and the Edinburgh Handedness Inventory Short Form (Veale, 2014) were used to assess a participants’ language background and handedness, respectively. The LHQ asked participants questions about their demographic information, native language, experience with other languages, and speaking, listening, reading, and writing proficiency in each language. The handedness inventory asked participants to rate how often they used their right versus left hand for writing, brushing their teeth, throwing, and using a spoon. For each item, they could select *always right* (100), *sometimes right* (50), *both equally* (0), *sometimes left* (−50), or *always left* (−100). Their scores for the four items were added up and divided by four to calculate each participant’s laterality quotient. Individuals with a laterality quotient of less than −60 are considered left-hand dominant while those with a quotient ranging from −60 to 60 have mixed-hand dominance. Right-handed individuals have a laterality quotient greater than 60.

Participants were also asked to report the amount of effort that they used when completing the tasks in the experiment on a scale from 1 “*least effort*” to 10 “*most effort*.” Effort was defined as how hard they tried rather than how well they did or how difficult they found the tasks. Overall, participants scored relatively high in their effort rating (*M* = 8.45, *SD* = 1.44), so we did not include the effort rating in our analyses.

### Procedure

All participants completed both the motor and language tasks as part of a larger battery of tasks that assessed motor and language production (not reported here). Participants provided written consent before beginning the experiment. All experimental procedures were approved by the Institutional Review Boards at both Penn State University (Approval No. 00008811) and University of Wisconsin-Madison (Approval No. 2013-1037-CR004). The experiment began with the first two arcs of the motor task, which always preceded the language task. Participants completed the last two arcs of the motor task after a number of intervening tasks not discussed in the current article. After completing the battery of tasks, participants filled out the LHQ and Edinburgh Handedness Inventory Short Form.

## Results

All mixed-effects models were conducted using the lme4 (Bates et al., 2015) and lmerTest (Kuznetsova et al., 2017) packages in R version 3.6.1 (R Core Team, 2016) with the *nloptwrap* optimiser and orthogonal contrasts for categorical variables. For each model, the maximal random-effects structure permitting model convergence was chosen. We systematically eliminated random effects of minimal theoretical relevance adhering to the guidelines specified by Barr et al. (2013) until convergence was achieved. Each model below specifies the final random-effects structure.

### Motor task production choice

**Figure 3.**
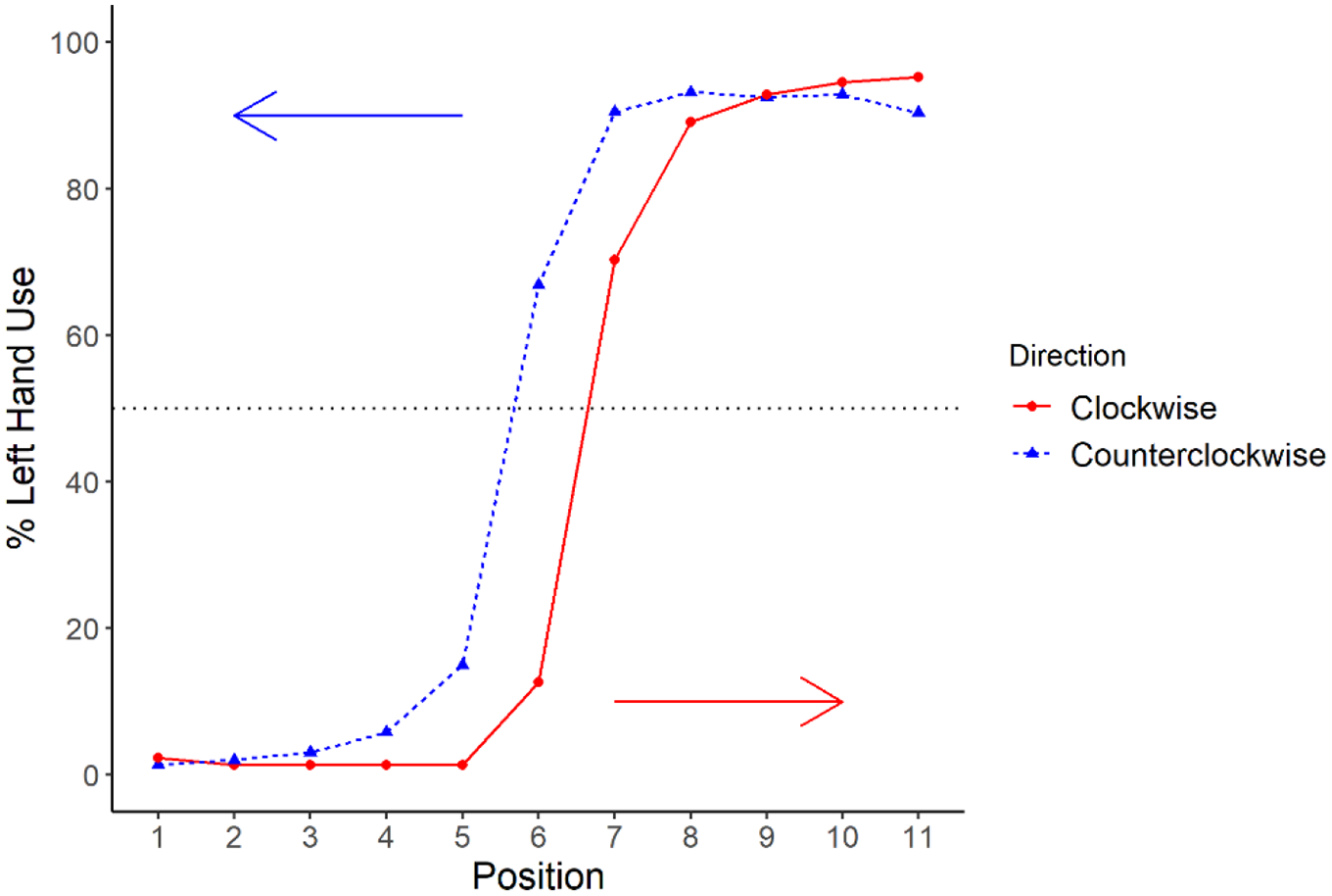
Percentage left-hand use per target position separated by arc progression. *Note.* The solid line represents behaviour on the clockwise progression, while the dotted line represents the counterclockwise progression. The horizontal line represents 50% left-hand use. Arrows indicate the order of target positions for each direction with trials beginning at Position 1 in the clockwise progression and Position 11 in the counterclockwise progression. Position 6 is the midline target in the arc.

To evaluate the presence of a hysteresis effect in participants’ performance on the motor task, we conducted a linear mixed-effects model with TP (the position where an individual switched hand use during an arc) as the outcome variable, the direction of the arc progression (clockwise or counterclockwise) as a within-subject fixed effect, and the participant’s condition (whether they completed the clockwise or counterclockwise progression first) as a between-subject fixed effect. We also included the interaction between direction and condition as a fixed effect. For random effects, we added a random intercept for each participant and a by-participant random slope for direction to the model. Figure 3 illustrates the difference in TP in the clockwise and counterclockwise arc progressions. We observed a significant main effect of direction on participants’ TP, β = −1.92, *t*(113.62) = −19.09, *p* < .001. The mean TP was at approximately Position 7 (7.18) in the clockwise direction and Position 5 (5.23) in the counterclockwise direction. There was no main effect of clockwise-first versus counterclockwise-first condition, β = −0.01, *t*(113.89) = −0.08, *p* = .94, nor a significant interaction between condition and direction, β = 0.03, *t*(113.62) = 0.15, *p* = .88.

**Figure 4.**
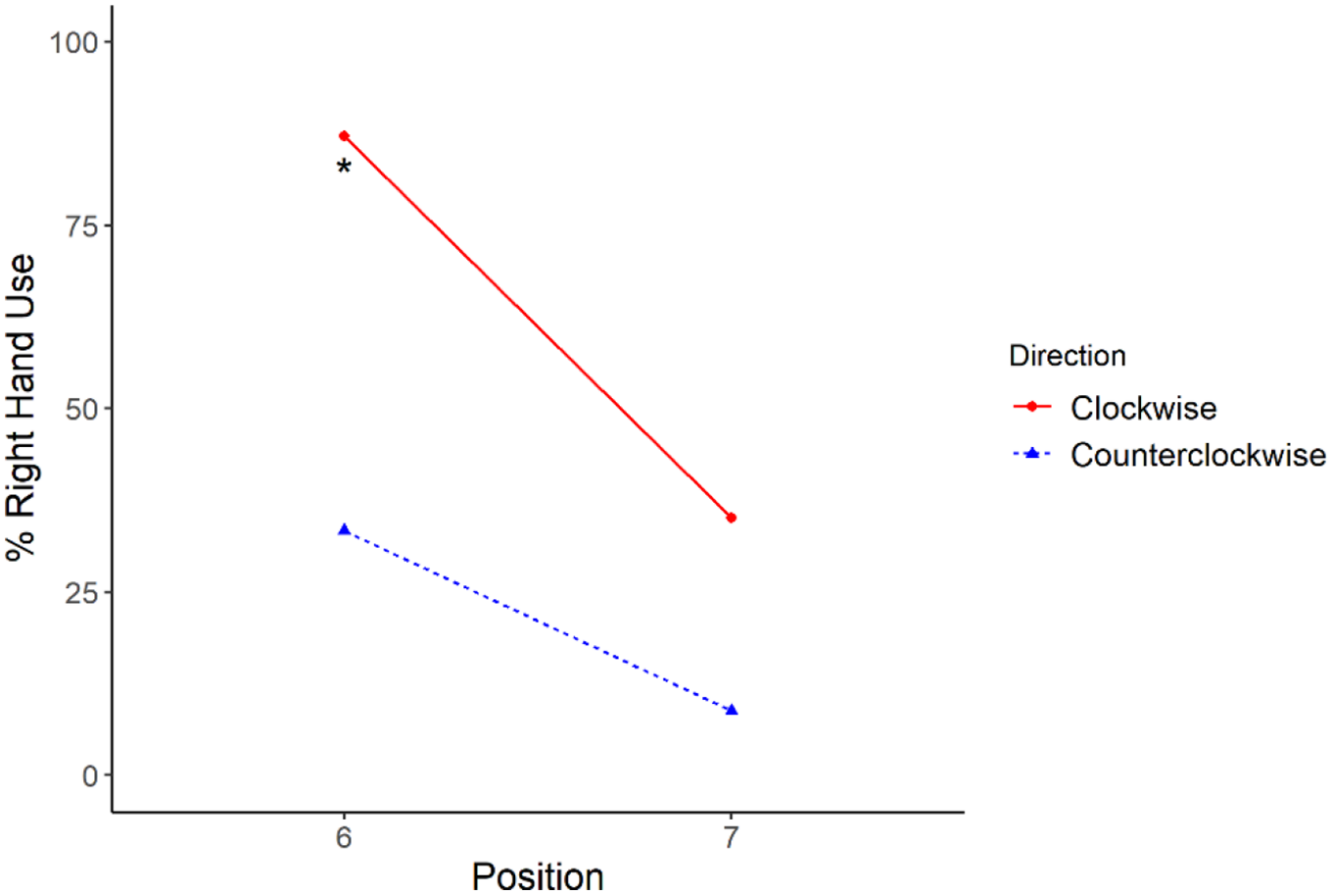
Percentage right-hand use at Positions 6 and 7. *Note*. The solid line represents behaviour on the clockwise progression while the dotted line represents the counterclockwise progression. The asterisk represents the percentage of right-hand use on the first practice trial. Position 6 represents the target at the midline of the display.

Our next analysis explored if the influence of prior history changed as a function of whether participants began the task with their dominant (as in the clockwise progression) or subordinate (as in the counterclockwise progression) hand. As noted above, prior to beginning the experimental task, participants engaged in an initial practice trial in which they touched a target located at the centre of the display. We focused on this central target because it required an equidistant reach for either hand and was thus roughly equated with respect to biomechanical requirements. Using this trial as an approximate baseline for touching the central target (Position 6) in the absence of recent production history, we compared this rate with the level of right-hand use at Position 6 in clockwise and counterclockwise progressions (see Figure 4). Specifically, we conducted a generalised linear mixed-effects model with hand use on the current trial (1 = *right hand*, 0 = *left hand*) as the outcome variable and trial type (first practice trial, Position 6 in the clockwise progression, or Position 6 in the counterclockwise progression) as a within-subject fixed effect. We also included a random intercept for each participant and random by-participant slope for trial type. There was a significant main effect of trial type on right-hand use (β = 4.65, *z* = 6.71, *p* < .001).

To follow-up the main effect, we conducted orthogonal contrasts comparing Position 6 in the clockwise progression to the first practice trial and Position 6 in the counterclockwise progression to the first practice trial. There was significantly more right-hand use at Position 6 in the clockwise progression (88.54%) relative to the first practice trial (82.11%; β = 1.41, *z* = 2.95, *p* = .003). Conversely, there was significantly less right-hand use at Position 6 in the counterclockwise progression (32.41%) than on the first practice trial (82.11%; β = 4.24, *z* = 9.15, *p* < .001).

To examine the simple effect size for each comparison, we calculated the odds ratio for these contrasts. Participants were approximately four times more likely to use their right hand at Position 6 in the clockwise progression relative to baseline (OR = 4.11, 95% CI = [1.57, 10.60]) and approximately 69 times more likely to use their right hand at baseline compared to Position 6 of the counterclockwise progression (OR = 69.29, 95% CI = [30.15, 191.64]). While we are unable to precisely estimate the magnitude of this effect given the width of our confidence interval, we can conclude that the effect is large since the minimum value for the odds ratio is approximately 30. This provides suggestive evidence that executing a planned reach with the subordinate hand may exert a more robust priming effect relative to dominant hand reaches (see “General Discussion” section).

### Language task production choice

**Figure 5.**
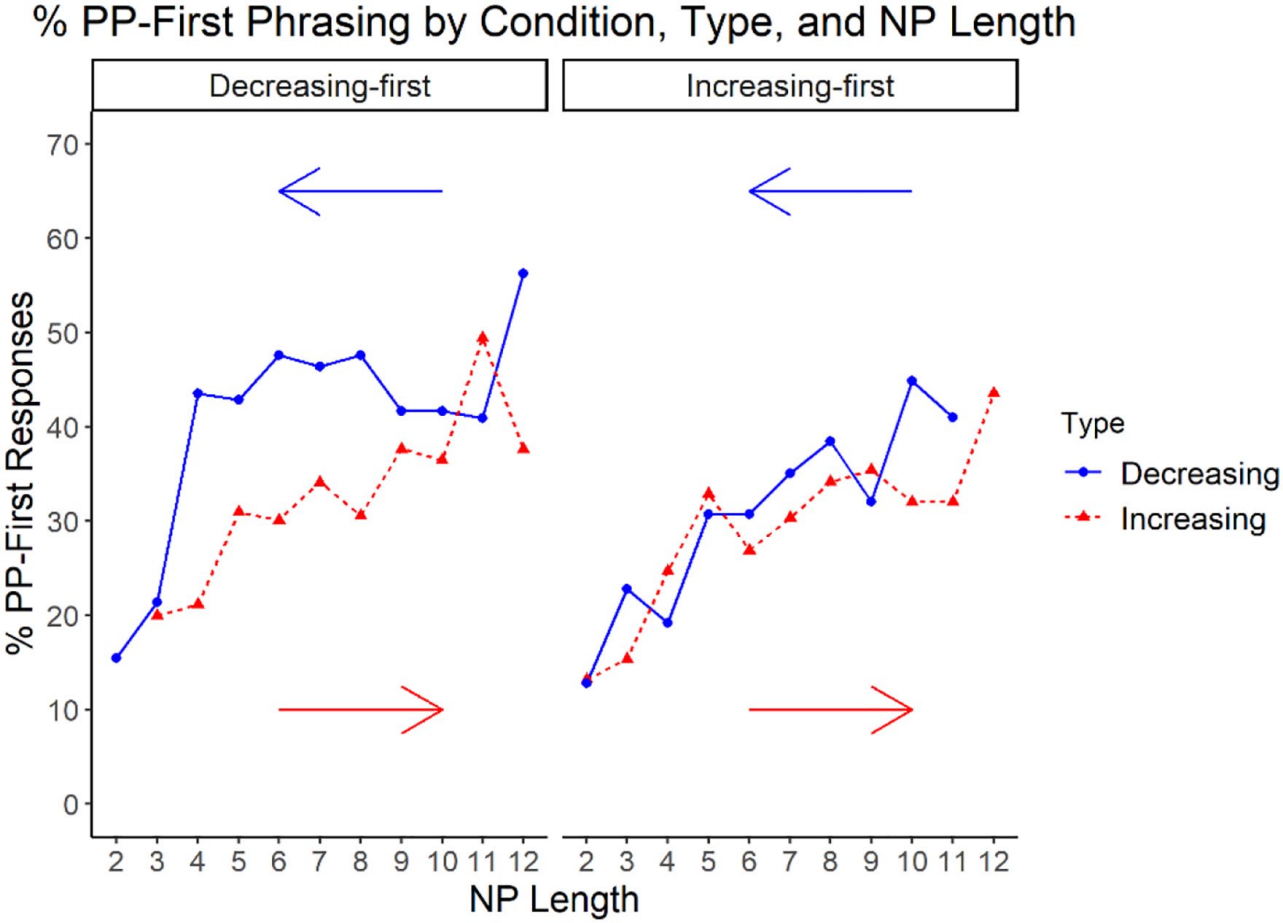
Percentage raw PP-first production per NP length separated by condition. *Note.* Trials increasing in NP length are represented by the dashed line and those decreasing in NP length are represented by the solid line. Arrows indicate the order of the NP lengths for each trial type (starting with NP length 2 on increasing trials and NP length 12 on decreasing trials).

On the motor task, almost all participants switched their hand use only once per arc which permitted TPs to be clearly identified. By contrast, the language task elicited greater variability and multiple switches in production choices (see Figure 5). Consequently, we added polynomial terms to the models of production choice on the language task as this afforded the ability to better identify TPs. Specifically, these TPs represented NP lengths (of a particular condition or trial type) in which the relationship between NP length and the probability of PP-first phrasing changed (e.g., switched from positive to negative). We utilised the ggeffects package (Lüdecke, 2018) to generate model predictions for polynomial terms.

Before conducting main analyses for the language data, we excluded data from trials with NP lengths 2 and 12 since participants in the Increasing-first condition had only one trial with NP length 12 and participants in the Decreasing-first condition had only one trial with NP length 2, making it impossible to compare production across trial type for these NP lengths. Thus, we included trials with NP lengths ranging from 3 to 11. Cubic was the highest-order polynomial term retained in the final model since model comparisons indicated that it contributed significantly more variance than the quadratic term for NP length in the language data, χ^2^(4) = 10.89, *p* = .03. By contrast, the quartic term did not contribute significantly more variance than the cubic term, χ^2^(4) = 3.72, *p* = .45.

**Figure 6.**
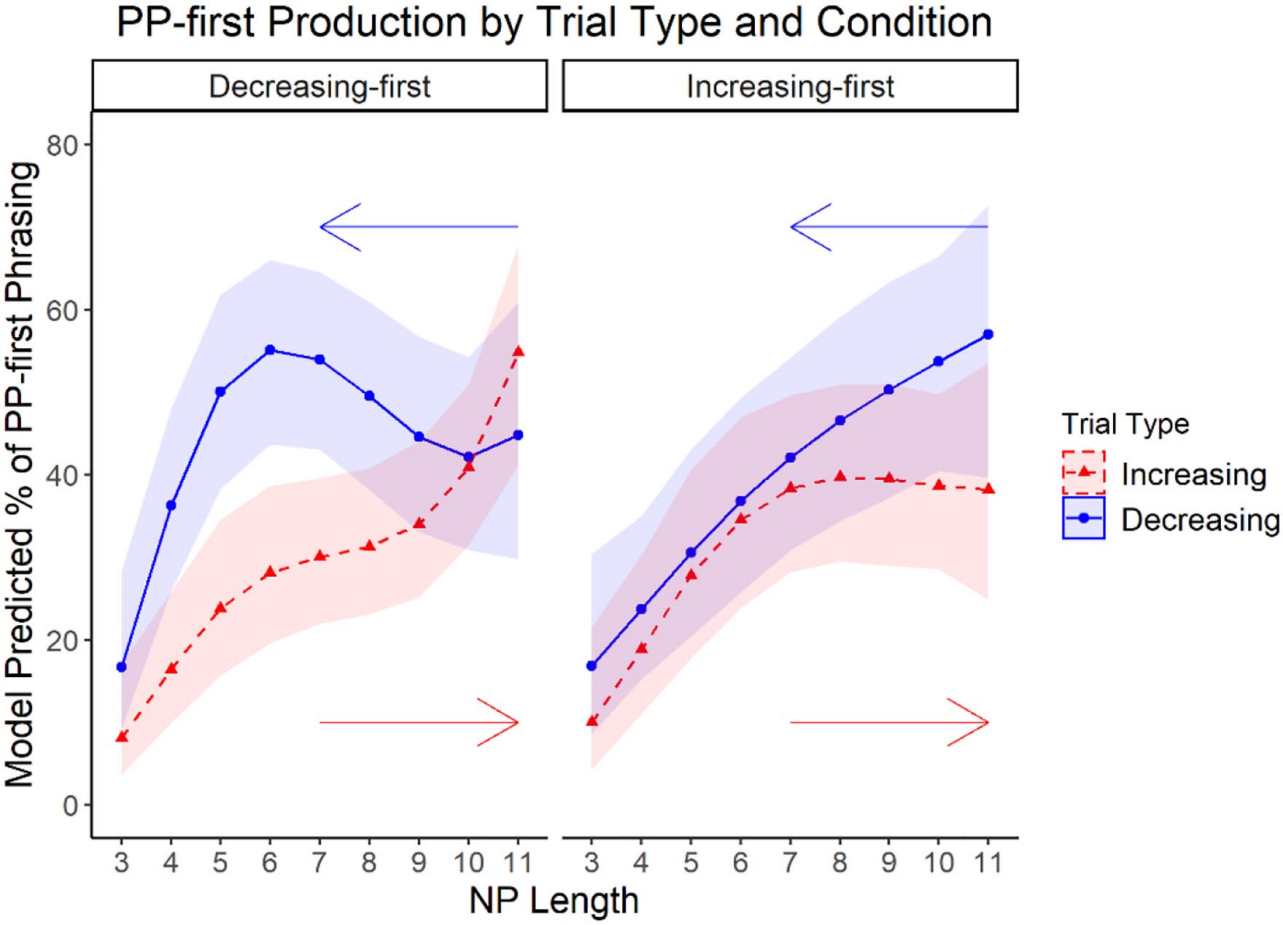
Percentage model predicted PP-first production per NP length separated by condition. *Note.* Trials increasing in NP length are represented by the dashed line and those decreasing in NP length are represented by the solid line. Arrows indicate the order of the NP lengths for each trial type (starting with NP length 3 on increasing trials and NP length 11 on decreasing trials).

We then conducted an omnibus test of the final model for the language data. There was a significant three-way interaction between the polynomial terms for NP length, and the other fixed effects of interest: trial type (increasing or decreasing) and condition, Increasing-first versus Decreasing-first; χ^2^(3) = 12.81, *p* = .005. This interaction suggested it was appropriate to evaluate the simple effects of the polynomial terms for the different subconditions of the language task (i.e., increasing trials in the Increasing-first condition, decreasing trials in the Increasing-first condition, etc.). Figure 6 illustrates the percentage of PP-first production predicted by the model separated by subcondition.

For increasing trials in the Increasing-first condition (represented by the dashed line on the right panel of Figure 6), there were significant effects for the linear (β = 22.87, *z* = 3.30, *p* < .001) and quadratic (β = −15.04, *z* = −2.81, *p* = .005), but not cubic (β = 4.12, *z* = .80, *p* = .43) terms for NP length indicating the existence of one TP. Participants in this subcondition began producing low levels of PP-first phrasing which increased steadily with NP length until reaching a length of seven words. On subsequent trials with longer NP lengths, PP-first production remained at roughly the level observed at NP length 7 rather than continuing to increase. A significant effect for the linear term (β = 27.80, *z* = 4.66, *p* < .001), though not the quadratic (β = −5.96, *z* = −1.17, *p* = .24) nor cubic terms (β = 1.33, *z* = .26, *p* = .79), was identified for decreasing trials in the Increasing-first condition indicating no TPs in the relationship between NP length and PP-first phrasing (see the solid line on the right panel of Figure 6). In this subcondition, the level of PP-first phrasing decreased steadily with decreasing NP length.

On decreasing trials for the Decreasing-First condition (the solid line on the left panel of Figure 6), participants exhibited significant effects for linear (β = 10.77, *z* = 2.02, *p* = .04), quadratic (β = −16.76, *z* = −3.66, *p* < .001), and cubic terms (β = 10.58, *z* = 2.34, *p* = .02) suggesting two TPs in the relationship between NP length and PP-first phrasing. The level of PP-first phrasing was roughly the same from NP lengths 11 to 10, but then steadily increased on subsequent trials until reaching an NP length 6. After this point, the level of PP-first phrasing decreased sharply on trials with NP lengths 3–5. On increasing trials in this condition (the dashed line on the left panel of Figure 6), there were significant effects for the linear (β = 31.57, *z* = 5.00, *p* < .001) and cubic terms (β = 10.13, *z* = 2.15, *p* = .03), but not the quadratic term (β = −4.68, *z* = −.95, *p* = .34) suggesting the existence of two TPs in the relationship between NP length and PP-first phrasing. The level of PP-first production increased steadily until NP length six, then increased at a slower rate between NP lengths 6 and 9, and finally increased sharply at NP lengths 10–11. In sum, we identified TPs and hysteresis areas in the relationship between NP length and PP-first production discussed further below.

### Motor task RT

We investigated whether RT decreased on trials with plan reuse for hand choice selection relative to hand switches. Before conducting main analyses, we standardised RTs within-subject by calculating z-scores to identify outliers and excluded RTs with a score lower than −3.00 or higher than 3.00. Even after excluding outliers, the RT data had a skewness of 2.08 indicating a significant departure from normality. To account for this, we log-transformed our RT data before conducting analyses.

We restricted the analysis to target Positions 4–8, as there was little variability in hand use at the more lateralised positions. This reflected our desire to test the influence of reuse at locations in which the biomechanical constraints did not dictate the choice, and this decision also resulted in a relatively equal number of trials with and without hand reuse (see Figure 3). Our linear mixed-effects model included log-transformed RT as the outcome variable and hand reuse (same vs different hand use as the previous trial), hand use on current trial (left vs right), direction of the current arc progression (clockwise vs counterclockwise), and target position (1–11, centred for the analysis) as within-subject fixed effects. We also included participant condition (which arc progression was completed first) as a between-subject fixed effect and an interaction between condition and direction of arc progression. For the random effects structure, we included a by-participant random intercept and a by-participant random slope for the direction of arc progression.

While we report coefficients obtained from analyses with log-transformed RTs, the reported means for all analyses and figures are based on raw RTs to facilitate clearer interpretation. There was a significant main effect of hand reuse, β = 0.02, *t*(2445) = 6.33, *p* < .001, with decreased RT on reuse (*M* = 1.07 s) compared to non-reuse trials (*M* = 1.13 s). In addition, participants exhibited significantly faster RTs when using their right hand (*M* = 1.05 s) relative to their left hand, *M* = 1.11 s, β = 0.01, *t*(2603) = 3.64, *p* < .001. There was also a significant main effect of position, β = 0.006, *t*(2557) = 4.50, *p* < .001, suggesting that RT increased for targets located further to right. This increase in RT is likely due to an increased reliance on left-hand use for reaching towards right-lateralised targets. There were no significant main effects of direction, β = −0.003, *t*(152.5) = −1.03, *p* = .31, and condition, β = 0.002, *t*(153.6) = 1.41, *p* = .16, and no significant interaction between direction and condition, β = −0.008, *t*(136.9) = −1.27, *p* = .21. Due to the incremental nature of the motor task, we also conducted analyses that focused on how RT differed at the TP, the position at which participants switched from using one hand to the other, relative to the surrounding target positions. The results of this analysis also suggest that hand reuse leads to RT savings (see the online Supplementary Material).

### Language task RT

Paralleling the motor task, we investigated whether RT would be decreased for trials with plan reuse (here referring to sequential productions of the same sentence structure) relative to switching. We extracted speech onset time (often termed initiation latency in language production) from audio recordings of the trials on the language task utilising modified Praat scripts (Boersma, 2001). A subset of trials was hand-coded by a trained research assistant as an index of reliability for the RTs automatically extracted by the Praat script. We excluded trials with an NP length that was less than six words from the RT analyses. This ensured that an observed reduction in RT for Plan Reuse trials was not solely driven by the reuse of NP-first phrasing on short NP length trials (which were far less variable in general than the higher NP lengths). This was analogous to the elimination of extreme target positions for the RT analyses of the motor task in that we wanted to have a relatively balanced comparison in the number of trials with and without reuse. We also removed RTs from error trials (i.e., trials with no response) and standardised RTs within-subject by calculating z-scores to identify outliers and excluded RTs with a score lower than −3.00 or higher than 3.00. Since the raw RT data had a skewness of 1.91 indicating a significant departure from normality, like the motor task, we log-transformed our RT data before conducting analyses.

We conducted a linear mixed-effects model with log-transformed RT as the outcome variable and trial type (increasing vs decreasing) and Plan Reuse on the current trial (different phrasing from previous trial vs same phrasing as previous trial) as within-subject fixed effects. We also included participant condition (Increasing-first vs Decreasing-first) as a between-subject fixed effect, and an interaction between trial type and condition as a fixed effect. In addition, we incorporated a random intercept for each participant.

There was a significant main effect of Plan Reuse on the current trial, β = −0.05, *t*(1838) = −4.77, *p* < .001. RT was significantly shorter on trials with Plan Reuse (*M* = 2.97 s) relative to those without (*M* = 3.83 s). There was no main effect of condition, β = −0.002, *t*(143.1) = −0.04, *p* = .97, but there was a significant main effect of trial type, β = 0.02, *t*(1790) = 2.71, *p* = .007, suggesting that RT was shorter for increasing trials (*M* = 3.12 s) relative to decreasing trials (*M* = 3.28 s). However, there was also significant interaction between condition and trial type, β = 0.10, *t*(1790) = 6.65, *p* < .001.

To follow-up the significant interaction between condition and trial type, we evaluated the simple effect of trial type in each condition. Participants in the Increasing-first condition exhibited significantly longer RT on increasing trials (*M* = 3.22 s) relative to decreasing trials, *M* = 3.06 s; β = −0.03, *t*(1790) = −2.73, *p* = .006. Conversely, participants in the Decreasing-first condition exhibited significantly longer RT on decreasing trials (*M* = 3.47 s) relative to increasing trials, *M* = 3.02 s, β = 0.07, *t*(1790) = 6.78, *p* < .001. This suggests that participants exhibited longer RTs on the first half of the trials during the language task relative to the second half as opposed to longer RTs on decreasing trials overall.

## General discussion

In this study, we sought to determine whether hysteresis effects observed in the motor domain find parallels in language production. To that end, participants completed a motor task and a language task that shared some features with the motor task. The motor task required participants to reach through an aperture to touch targets appearing on a touchscreen. After every trial, the targets incremented by one position in either a clockwise or counterclockwise progression. The corresponding language task asked participants to construct sentences aloud by ordering two phrases (a NP and a PP) following a subject-verb phrase presented visually on a computer screen. Similar to the incremental nature of the motor task, the length of the NP increased or decreased by one word on each trial, depending on condition.

We found evidence for three parallels across the two tasks. First, we observed hysteresis asymmetries in production choices based on the direction of the incremental progression (clockwise or counterclockwise in the motor task, increasing or decreasing NP length in the language task). We further observed that the impact of prior history on production varied as a function of response preference in both tasks, providing evidence for inverse preference effects comparable to those found in syntactic priming (Ferreira & Bock, 2006). Finally, we observed evidence for RT savings on trials with Plan Reuse in both tasks, consistent with computational efficiency accounts of hysteresis (Rosenbaum & Jorgensen, 1992; Valyear et al., 2018). Despite the difficulty of balancing task demands across domains, these parallels suggest that hysteresis effects manifest in motor and language planning.

### Production choice asymmetry

In both the motor and language production tasks, we observed a signature of hysteresis, namely an asymmetry in production choices as a consequence of production history. In both tasks, the TPs in production choice differed across subconditions. These TPs may be thought of as a hysteresis area (see Weiss & Wark, 2009). That is, the points along the continuum at which the production choice was most susceptible to the effects of production history.

Since the task demands and complexity differed across domains, these TPs manifested differently and required different analyses. In the motor task, we found significant hysteresis effects in both the clockwise and counterclockwise directions. Participants exhibited a single TP that varied as a function of where the trial sequence started (to the left or right of the participant). Specifically, the mean TP in the clockwise progression occurred at approximately Position 7, whereas in the counterclockwise progression, it was approximately at Position 5. This was largely comparable to the effects observed with nonhuman primates using an analogous task (Weiss & Wark, 2009).

Due to the biomechanical constraints imposed by the hoop aperture, both left- and right-hand use approached 100% at the extreme target positions. This was intentional, as we were concerned that without any constraints, our right-hand dominant participants would reach with their right hand every trial as that would impose fewer cognitive and biomechanical demands for execution (Janssen et al., 2009, 2011; Sainburg, 2002; Schütz & Schack, 2020a). We surmised the hoop aperture would encourage participants to switch hands at some point during the continuum (though as several participants demonstrated, in principle, it was possible to use only one hand for every position; see also Weiss & Wark, 2009).

By contrast, in the language task, there were no analogous biomechanical constraints on production and, consequently, production of either form was probabilistic even at the extremes. Moreover, participants switched from one production choice to the other throughout each progression. Only the dominant production choice (NP-first phrasing) approached ceiling level production (86%) at the shortest NP lengths. At the other end of the continuum, production choices tended to be more evenly distributed, consistent with prior studies of HNPS (Stallings et al., 1998). The subordinate form, PP-first phrasing, reached a maximum of nearly 63% production at an NP length of 12 for participants in the Decreasing-first condition, underscoring the prevalence of NP-first phrasing overall.

Despite the lack of uniform responses at either end of the continuum, we identified TPs in the language data by incorporating polynomial terms into our analyses which better captured the nonlinear relationship between NP length and PP-first phrasing. Analogous to the motor task, the TPs on the language task varied as a function of whether the trial sequence began with short or long NPs across condition and trial type (see Figure 5). On increasing trials in the Increasing-first condition, PP-first production increased with NP length until plateauing at NP length 7. Conversely, there was no overt TP for decreasing trials. In the Decreasing-first condition, there were TPs at NP lengths 6 and 10 for decreasing trials and 6 and 9 for increasing trials. Interestingly, while PP-first phrasing increased with NP length between TPs on increasing trials, it increased as NP length *decreased* between TPs on decreasing trials. These asymmetries in language production suggest that hysteresis effects, as they have been described previously in the motor literature (e.g., Rosenbaum & Jorgensen, 1992; Rostoft et al., 2002; Schütz & Schack, 2020a etc.) also occur in language production when the tasks share incremental design features.

### Inverse preference effects

As noted in the “Introduction” section, less frequent forms tend to exhibit more robust priming effects in language production (Ferreira & Bock, 2006; Segaert et al., 2016). This pattern was affirmed in our language task, particularly in the Decreasing-first condition that started with long NPs. As mentioned in the previous section, overall PP-first production actually increased even as the NPs progressively became shorter, specifically from NP length 10 to NP length 6. This is the exact opposite of what has typically been reported for these NP lengths in natural language corpora and in tasks designed to limit priming effects (Hawkins, 1994; Stallings et al., 1998).

We argue that the increase in PP-first phrasing with decreasing NP length is an example of the *inverse preference effect*. Participants in the Decreasing-first condition produced the largest proportion of sentences using the subordinate form (PP-first) at the onset of their session (at NP lengths 12 through 10). The influence of these production choices may have permeated the successive trials despite the decreasing NP length. This contrasts with the Increasing-first condition in which participants had already produced a mix of NP-first and PP-first choices, with mostly NP-first phrasing at the lower weights. Overall, our findings are certainly consistent with the notion that producing the subordinate or less frequent form at the onset resulted in a protracted impact on production choices in a way that was not observed when the progressions began with the more frequent form (NP-first phrasing in the Increasing-first condition).

We also found suggestive evidence of an analogous inverse preference effect in the motor task. In the practice trials prior to starting the motor task, participants were asked to touch a dot that approximately corresponded to Position 6 in the progression. Roughly 80% of participants used their right hand, suggesting that the point of subjective equality (between right- and left-hand use) was likely farther to the participant’s right (Figure 4; see also Schütz & Schack, 2020a for evidence of shifted points of subjective equality due to hand dominance). This was unsurprising given the right-hand dominance of participants. Yet, when participants completed a counterclockwise progression, they persisted in using their *left hand* in 68% of trials at Position 6. Even Position 7 on the clockwise progression did not evidence an effect of this magnitude. This suggests a possible inverse preference effect in hand use with greater priming resulting from previous subordinate (left) relative to dominant (right) hand use.

Models of syntactic priming differ in how they account for the inverse preference effect (Chang, 2009; Chang et al., 2006; Cho et al., 2020; MacDonald, 2013; Reitter et al., 2011), though several borrow components from models of action production (e.g., Anderson et al., 2004; Botvinick & Plaut, 2004; Pomerleau, 1991). This overlap suggests an anticipation of domain-general effects of reuse, such as those reported here. It has recently been argued that activation-based theories may be better suited to account for self-priming effects, which arguably are prevalent in both domains (see Cho et al., 2020). While our findings cannot adjudicate between alternative models, we see the current study, with its focus on commonalities across quite different domains, as a useful step in further understanding changes in behaviour that can be described as priming or hysteresis.

### RT savings

The computational efficiency account of hysteresis suggests that reusing recently deployed plans carries less cost relative to creating a new plan from scratch (Rosenbaum & Jorgensen, 1992; Valyear et al., 2018). This has been evidenced by reduced RTs for previously used production choices in both language (e.g., Smith & Wheeldon, 2001) and motor experiments (e.g., Schütz & Schack, 2020a; Valyear & Frey, 2014, 2015; Valyear et al., 2018). Correspondingly, we found that participants exhibited shorter RTs across both domains on self-primed trials relative to those in which they switched hands or sentence types. This was true across the different conditions and trial types on both tasks. The RT savings observed on reuse trials also did not vary as a function of left-hand compared to right-hand reaches on the motor task. These cross-domain results are generally consistent with approaches to reuse as emergent from quite general memory efficiency constraints (Dasgupta & Gershman, 2021).

## Concluding remarks

While our study was successful in demonstrating similar effects across motor and language production, our findings also underscore two related challenges confronting cross-domain comparisons. One critical concern is equating the units of analysis across tasks. Both the language and motor literature grapple independently with reaching a consensus on what units constitute fundamental primitives, and that compounds the complexity of this issue. For example, in the language literature, the psychological reality of the fundamental units of composition has been a source of continued debate (e.g., with respect to phonemes, see Foss & Swinney, 1973; Samuel, 2020; Savin & Bever, 1970). Likewise, in the motor literature, researchers continue to grapple with the primitives of motor control (e.g., Hogan & Sternad, 2012; Latash, 2020). This relates to the second challenge of this approach, namely equating the level of planning complexity across tasks. As we encountered in our previous work (Koranda et al., 2020), it is much easier to identify the conceptual effects of interest (here hysteresis), but considerably more difficult to equate the level of planning in designing experimental tasks. To wit, our findings in the motor task yielded far less variability than the language task, and this precluded an analysis of individual differences in performance across tasks and also yielded data that required different analytic approaches for each task. Nevertheless, it is all the more remarkable that we find many parallels across tasks despite these acknowledged challenges.

## Supplemental Material

sj-docx-1-qjp-10.1177_17470218221094568 – Supplemental material for Hysteresis in motor and language production Click here for additional data file. Supplemental material, sj-docx-1-qjp-10.1177_17470218221094568 for Hysteresis in motor and language production by Amy L Lebkuecher, Natalie Schwob, Misty Kabasa, Arella E Gussow, Maryellen C MacDonald and Daniel J Weiss in Quarterly Journal of Experimental Psychology
