# Supplementary material for: Hysteresis in motor and language production
Source: Q J Exp Psychol (Hove). 2022 May 18;76(3):511–27. doi: 10.1177/17470218221094568 (PMC9936447; doi:10.1177/17470218221094568)
Supplement: sj-docx-1-qjp-10.1177_17470218221094568 – Supplemental material for Hysteresis in motor and language production [file sj-docx-1-qjp-10.1177_17470218221094568.docx]

Supplementary Material for:

**Hysteresis in Motor and Language Production**

Amy L. Lebkuecher, Natalie Schwob, Misty Kabasa, Arella E. Gussow, Maryellen C. MacDonald, & Daniel J. Weiss

**Supplemental Motor RT Analysis**

In addition to evaluating RT savings based on hand reuse, we also conducted analyses that focused on how RT differed at the transition point (TP), the position at which participants switched from using one hand to the other, relative to the surrounding target positions. We included the TP, two positions prior (TP-1, TP-2), and two positions after the transition point (TP+1, TP+2) in the analysis. This approach is in line with previous findings of an increase in RT on the trial immediately preceding a switch in hand use on a similar incremental task (Schütz, & Schack, 2020b), suggesting that the binary categorization of trials according to reuse and non-reuse may mask some RT patterns.

The linear mixed effects model was identical to the primary model of RT on the motor task in terms of the outcome variable, fixed and random effects structure with one exception. We replaced the fixed effect of hand reuse with trial type (TP-2, TP-1, TP, TP+1, TP+2). Mean raw RTs at TP-2, TP-1, TP, TP+1 and TP+2 are illustrated in Figure 7. There was a significant main effect of trial type (*β* = .005, *t*(2403) = 14.79, *p* < .001). Additionally, participants exhibited significantly shorter RT when using their right hand (*M* = 1.05s) relative to their left hand (*M* = 1.11s, *β* = -.002, *t*(2403) = -4.59, *p* < .001). There was also a significant main effect of position (*β* = .0003, *t*(2403) = 2.06, *p* = .04) suggesting that RT increased for targets located further to right. As in the primary analysis of RT on the motor task, this increase in RT is likely due to an increased reliance on left hand use for reaching towards right-lateralized targets.

There were no significant main effects of direction (*β* = -.001, *t*(147.1) = -1.59, *p* = .11) and condition (*β* = .002, *t*(152.7) = 1.73, *p* = .08) and no significant interaction between direction and condition (*β* = .0002, *t*(139.9) = -.33, *p* = .74). To follow-up the significant main effect of trial type, we contrasted RT at TP relative to TP+1 and TP+2 as well as RT at TP-1 relative to TP-2 and TP+2. RT significantly increased at TP (*M* = 1.13s) relative to TP+1 (*M* = 1.04s) and TP+2 (*M* = 1.02s, *β* = .04, *t*(2403.00) = 8.61, *p* < .001). Since participants used the same hand on TP, TP+1 and TP+2, the shorter RT displayed on hand reuse (TP+1, TP+2) relative to hand switch (TP) trials provided evidence for a computational efficiency account of hysteresis (Valyear et al., 2018). RT was also significantly increased at TP-1 (*M* = 1.15s) relative to TP-2 (*M* = 1.05s) and TP+2 (*M* = 1.02s, *β* = .05, *t*(2403.00) = 10.78, *p* < .001).

The results of this analysis largely mirrored the findings of increased RT when switching hand use from the primary analysis (i.e., at the TP), but suggested that RT also increased at TP-1 despite hand reuse on this trial. Consistent with previous findings of an increase in RT immediately preceding a switch in hand use on a similar incremental task (Schütz, & Schack, 2020b), we propose that the increased RT at both TP and TP-1 reflected a hysteresis area, here manifest as a zone of uncertainty in hand use. Valyear and colleagues (2018) suggested that when the biomechanical costs of left- versus right-hand use are roughly equivalent, it is more likely that recent production history will influence current hand choice. In our task, the biomechanical costs of left- versus right-hand use were more comparable for central target positions than at either of the extremes, and thus we observed a hysteresis area in these central locations, as reported in prior studies (Schütz & Schack, 2020b; Valyear et al., 2018; Weiss & Wark, 2009). Schütz and Schack (2020b) suggested that the sequential nature of their task allowed participants to predict the location of objects in advance. Thus, the design of the motor task in our study current study may also have elicited anticipatory planning of hand use on upcoming trials. On this view, the increase in RT prior to the switch point reflects greater uncertainty regarding hand selection as a result of anticipating the upcoming transition. After the change in hand selection occurred, RT decreased again (consistent with the reuse effects mentioned above). Due to the much larger number of switches in the language production task, we could not conduct a parallel analysis for those data.

**Figure 7**

*Mean RT in Seconds by Trial Type*

**
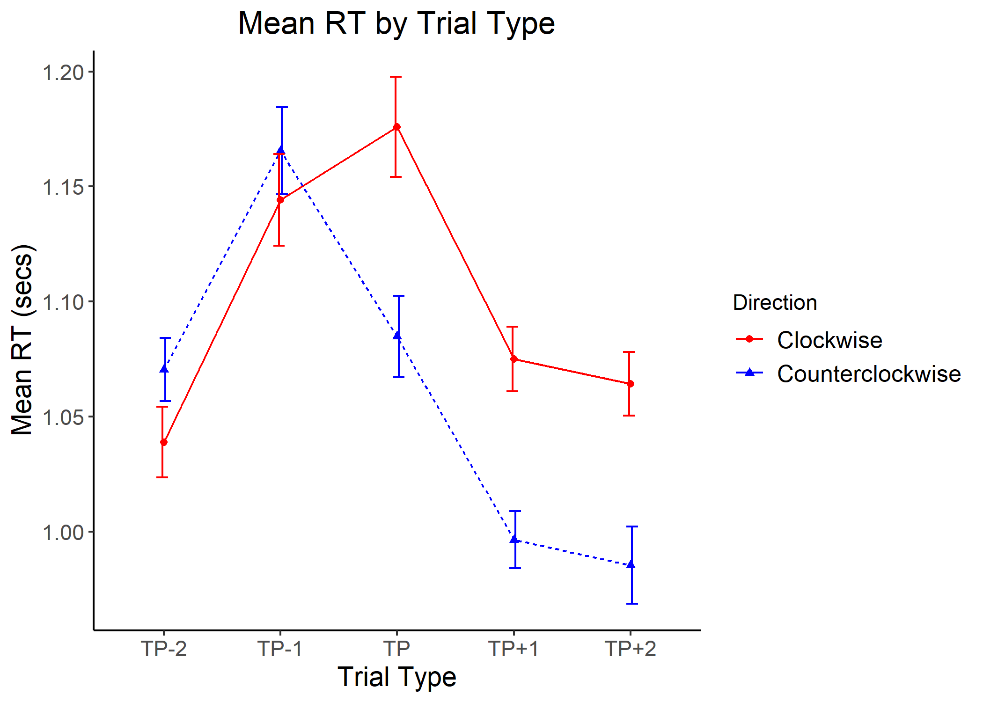
**

*Note.* The solid line represents mean RT in the clockwise arc progression and the dashed line represents the counterclockwise arc progression. The vertical bars represent the standard error of the mean.
